# Endoplasmic Reticulum Protein Targeting of Phospholamban: A Common Role for an N-Terminal Di-Arginine Motif in ER Retention?

**DOI:** 10.1371/journal.pone.0011496

**Published:** 2010-07-09

**Authors:** Parveen Sharma, Vladimir Ignatchenko, Kevin Grace, Claudia Ursprung, Thomas Kislinger, Anthony O. Gramolini

**Affiliations:** 1 Department of Physiology, University of Toronto, Toronto, Ontario, Canada; 2 Ontario Cancer Institute, University Health Network, Toronto, Ontario, Canada; 3 Department of Medical Biophysics, University of Toronto, Toronto, Ontario, Canada; 4 Division of Cellular and Molecular Biology, Toronto General Research Institute, Toronto, Ontario, Canada; Purdue University, United States of America

## Abstract

**Background:**

Phospholamban (PLN) is an effective inhibitor of the sarco(endo)plasmic reticulum Ca^2+^-ATPase, which transports Ca^2+^ into the SR lumen, leading to muscle relaxation. A mutation of PLN in which one of the di-arginine residues at positions 13 and 14 was deleted led to a severe, early onset dilated cardiomyopathy. Here we were interested in determining the cellular mechanisms involved in this disease-causing mutation.

**Methodology/Principal Finding:**

Mutations deleting codons for either or both Arg13 or Arg14 resulted in the mislocalization of PLN from the ER. Our data show that PLN is recycled via the retrograde Golgi to ER membrane traffic pathway involving COP-I vesicles, since co-immunoprecipitation assays determined that COP I interactions are dependent on an intact di-arginine motif as PLN RΔ14 did not co-precipitate with COP I containing vesicles. Bioinformatic analysis determined that the di-arginine motif is present in the first 25 residues in a large number of all ER/SR Gene Ontology (GO) annotated proteins. Mutations in the di-arginine motif of the Sigma 1-type opioid receptor, the β-subunit of the signal recognition particle receptor, and Sterol-O-acyltransferase, three proteins identified in our bioinformatic screen also caused mislocalization of these known ER-resident proteins.

**Conclusion:**

We conclude that PLN is enriched in the ER due to COP I-mediated transport that is dependent on its intact di-arginine motif and that the N-terminal di-arginine motif may act as a general ER retrieval sequence.

## Introduction

Sarco(endo)plasmic reticulum Ca^2+^-ATPases (SERCAs) are 110-kDa membrane proteins that transport Ca^2+^ from the cytosol actively to the lumen of the sarco(endo)plasmic reticulum. In cardiac muscle, SERCA2a can associate with a 52-amino acid transmembrane phosphoprotein, phospholamban (PLN) [1]. In its dephosphorylated form, PLN interacts with SERCA2a to inhibit Ca^2+^ transport by lowering the apparent affinity of SERCA2a for Ca^2+^: upon PKA-mediated phosphorylation of PLN, its inhibitory effect on SERCA2a is relieved [2]. The ability of PLN to regulate SERCA2a activity, thereby regulating the rate of cardiac relaxation and the size of the SR Ca^2+^ store, makes PLN a crucial regulator of cardiac function [3]. Recently, a mutation of PLN in which one of the N-terminal di-arginine residues at positions 13 and 14 was deleted led to a severe, early onset dilated cardiomyopathy [4]. In fast twitch skeletal muscle SERCA1a associates with sarcolipin (SLN), a 31-amino acid protein which is an effective inhibitor of the SERCA molecule [5]–[7]. PLN and SLN share significant amino acid sequence identity and gene structure and are clearly homologous members of a gene family [5], [8]. We have previously reported that the RSYQY amino acid sequence at the C-terminus of SLN is vital in the retention of SLN in the ER/SR membrane [9]. The deletion of this sequence results in the mislocalization of SLN. However, the lack of this sequence in PLN implied that the retention of PLN in the ER/SR membrane is conducted by a different mechanism.

Two distinct mechanisms for maintaining and concentrating proteins in the ER have been well defined: (*1*) ER proteins could be retained by active exclusion from vesicles that exit the ER, or (*2*) ER proteins exit the ER, but are subsequently retrieved from a post-ER compartment via a retrograde transport flow [10]. Concerning ER retrieval, the well characterized KDEL motif in the C-terminal region of soluble ER proteins, together with the KDEL receptor protein, Erd2p, has been shown to mediate the retrieval of luminal ER proteins from the Golgi apparatus [11]. In addition, several families of transmembrane ER proteins have been shown to have an ER retention signal consisting of a di-lysine motif, KKxx or xKxK, located near the C terminus. In these cases, lysine must be positioned three residues from the C-terminus and the second residue must be present either four or five residues from the C-terminus [12]. These lysines have been shown to form part of an interaction site with coatomer protein I (COP-I) [10], [13], [14]. The role and mechanism of the N-terminal di-arginine motif xRRx, in ER retention is less established. However, the di-arginine motif has been demonstrated to be essential for correct expression of the plasma membrane K^+^ (ATP)-sensitive potassium channel. Functional K^+^-ATP channels exists as octamers, but the subunits are expressed and retained in the ER by a C-terminal di-arginine motif. Only after the assembly of the complete octamer and the masking of the di-arginine motif is the K^+^-ATPase transported to the plasma membrane [15], [16].

In the present study, we describe evidence showing that the di-arginine motif, located near the N-terminus of PLN at positions 13 and 14, may be responsible for ER enrichment of PLN. A greater understanding of the role of this domain in regulating PLN in cardiac muscle appears further warranted, given our recent finding that a deletion in the human PLN gene, deleting arginine 14 (RΔ14) results in lethal hereditary cardiomyopathy [4]. We performed site-directed mutagenesis to generate arginine motif mutants and analyzed the subcellular localisation of PLN and these mutants. Here we report that mutation or deletion of either arginine in the di-arginine motif results in mislocalization of PLN from the ER.

## Results

### Mutation in di-arginine motif causes mislocalization of PLN

**Figure 1:**
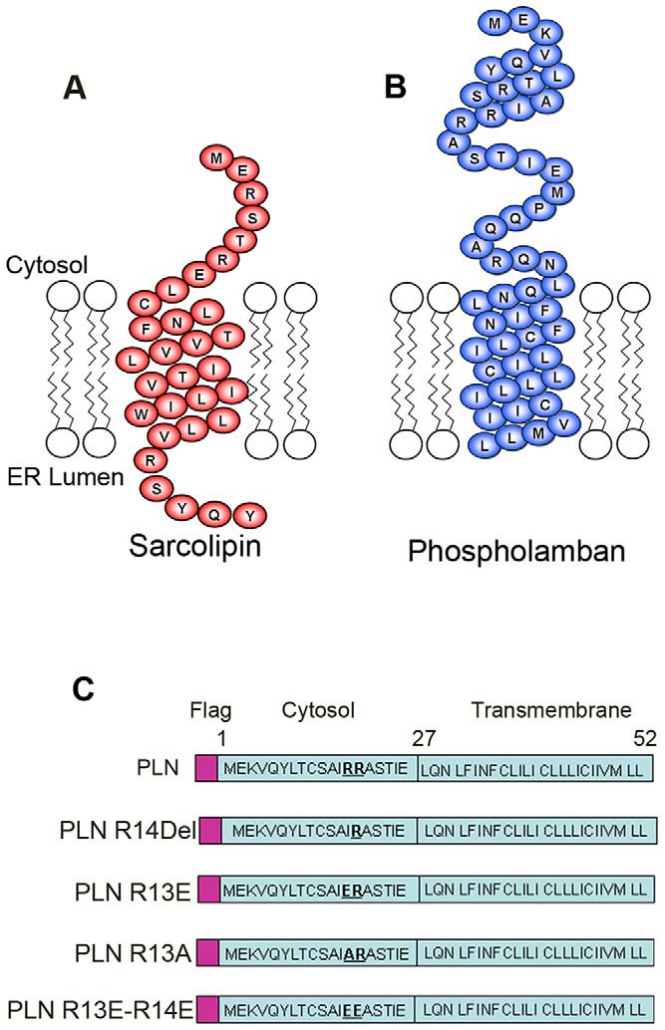
Schematic representation of SLN, PLN and PLN expression constructs. Model of rabbit (**A**) SLN and (**B**) PLN amino acid sequence. Note PLN has di-arginine residues in its N-terminal but a lack of C-terminal luminal domain as seen in SLN. (**C**) PLN expression constructs were generated with an N-terminal FLAG epitope. NF-PLN point and deletion mutants were generated subsequently by site directed mutagenesis.

**Figure 2:**
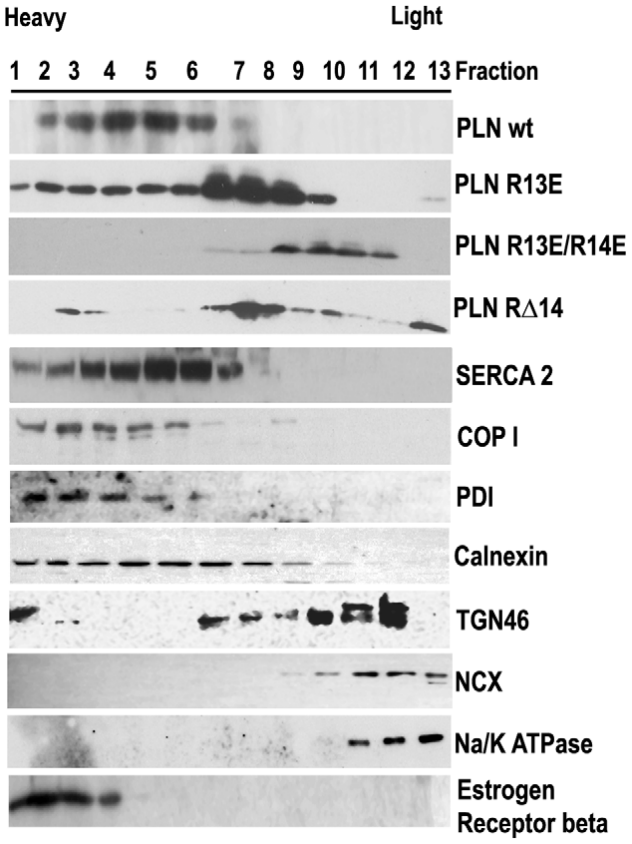
PLN arginine mutants are not retained in the ER. Subcellular fractionation of wild type NF-PLN, and di-arginine motif PLN mutants; RΔ14 PLN, R13E and R13E/14E. HEK cells were transiently transfected with tagged WT-PLN or PLN mutants and cell lysates run on a continuous 20–60% sucrose gradient. Fractions 1–13 contain the highest to lowest sucrose concentrations respectively. ER markers SERCA2a, coatomer protein I (COP-I), protein disulfide isomerase (PDI) and calnexin as well as plasma membrane protein markers Na/K ATPase and Na/Ca exchanger (NCX), a nuclear protein, estrogen receptor beta, and a trans golgi network marker (TGN46) were detected as markers to monitor the fractionation procedure. A minimum of 3 experiments were performed for each gradient.

PLN and SLN share significant amino acid sequence homology (**Figure 1A and B**). We have previously shown that the RSYQY amino acid sequence at the C-terminus of SLN is vital in the retention of SLN in the ER/SR membrane [9]. The lack of a known ER retention sequence in PLN and the finding that the deletion of arginine 14 causes lethal human cardiomyopathy led us to create a PLN RΔ14 construct as well as a series of constructs that contain mutations in one or both arginine residues (**Figure 1C**). Subcellular fractionation by sucrose density gradients is a well established technique for separating different organelles [17], [18]. We used a continuous sucrose gradient to examine PLN trafficking upon mutation of the di-arginine motif. HEK cells were transiently transfected with plasmids expressing either an intact di-arginine motif (WT) or one of three constructs containing a mutated di-arginine motif (PLN RΔ14, PLN R13E or PLN R13E/R14E) (see Fig. 1). Lysates were separated on a 20–60% sucrose gradient (as described in materials and methods) and 13 equal volume fractions were collected and analysed by immunoblotting (**Figure 2**). Wildtype PLN, with an intact di-arginine motif, eluted in fractions 1–7. SERCA2a, the major protein of the sarcoplasmic reticulum and the protein most likely to interact with wt PLN [19], [20], was also found in fractions 1–7. Coatomer protein I (COP-I), a protein involved in the retrieval of membrane proteins to the ER was found in lanes 1–8. We analysed two other known resident ER proteins, calnexin [21] which showed staining in lanes 1–8, and protein disulfide isomerase (PDI) [22] showed staining in lanes 1–6, thereby demonstrating that ER proteins are eluted in the heavier fractions of the sucrose density gradient. The estrogen receptor beta, a known nuclear membrane protein [23] eluted in lanes 1–4. The elution profiles of the PLN di-arginine mutants, however, differed considerably from wildtype PLN. PLN R13E showed some elution in fractions 1–6 but more than 50% of PLN R13E eluted in fractions 7–10. The double PLN mutant (PLN R13E/14E) only eluted in fractions 7–12. The profile of PLN RΔ14 was less uniform than wildtype PLN with small amounts eluted in fraction 3, while the bulk of the mutant eluted in fractions 6–10 indicating a significant shift towards lighter fractions. In fact, more than 15% of mutant PLN eluted in fractions 10–13, where the Na/Ca exchanger and the Na/K ATPase, two known plasma membrane proteins [24], were also seen to elute. The elution profiles of both PLN R13E and PLN R13E/14E showed overlap with the trans golgi protein 2 (TGOLN2; also known as TGN46) which eluted in fractions 7–12. TGN46 is a marker of trans golgi network [25] and its elution profile overlaps in part with the profiles of the plasma membrane markers Na/Ca exchanger and the Na/K ATPase.

**Figure 3:**
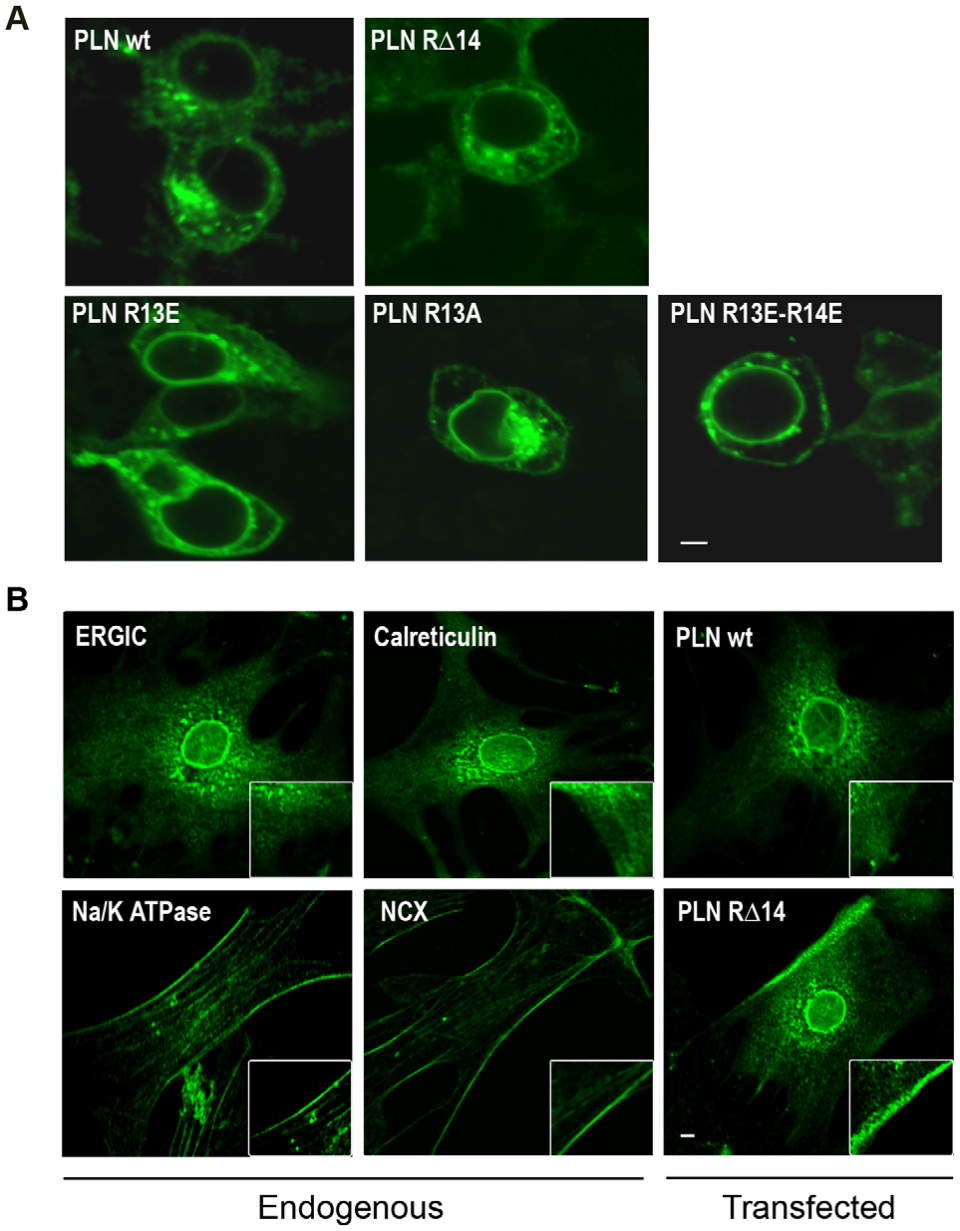
PLN arginine mutants are mislocalized out of the ER. (**A**) HEK cells were transiently transfected with tagged WT-PLN or PLN mutants and visualized by confocal microscopy to detect the flag tag. Note that mutation of either Arg13 or Arg14, or both together, resulted in mislocalization of PLN out of the ER with significant expression at the plasma membrane. Cells shown represent at least 20 representative cells per condition. (**B**) Primary mouse fibroblasts were transiently transfected with tagged WT-PLN or the RΔ14 PLN construct and visualized by confocal microscopy. Endogenous ER markers ERGIC-53 and calreticulin as well as plasma membrane protein markers Na/K ATPase and Na/Ca exchanger (NCX) were probed against to allow accurate visualization of the ER and the plasma membrane. WT-PLN shows staining similar to the ER markers and RΔ14 PLN shows more diffuse ER staining as well as pronounced plasma membrane staining. Inserts shows a higher magnification of the cell membrane. Note, no plasma membrane staining is seen in WT-PLN and the other ER proteins but clear membrane staining is seen for RΔ14 PLN and the plasma membrane proteins NCX and Na/K ATPase. (Scale bar, 10 µm)

To visualize the subcellular localization of wildtype and mutant PLN constructs, we performed confocal microscopy examining transiently transfected cells HEK-293 cells and larger, primary fibroblasts. In HEK-293 cells (**Figure 3A**) wildtype NF-PLN showed a clear perinuclear localization together with a highly organized web-like distribution throughout the cell (**Figure 3A**); presented a well defined and typical staining pattern of ER targeted proteins, as demonstrated previously [9]. Similarly, well defined ER staining patterns were also seen in the primary fibroblasts (**Figure 3B**) as exemplified by the staining of the known ER resident proteins calreticulin [26] and ERGIC-53 [27] in primary fibroblasts (**Figure 3B**). However, the PLN constructs containing mutations in the di-arginine motif show disrupted ER staining patterns in both HEK cells and fibroblasts (Fig. 3A and 3B). Specifically, the R13E and R13A mutants have a more punctate staining pattern with large aggregates found close to the nuclear region, along with some evidence of plasma membrane staining, which was never observed in wildtype PLN-transfected cells (**Figure 3A**). This was also true for the double mutant R13E/R14E and the PLN RΔ14 construct which showed the same punctate staining with a significant amount of expression found in the plasma membrane (**Figure 3A**). Similar diffuse cytosolic staining and pronounced plasma membrane staining patterns were observed in the SLN mutants with disrupted ER sorting sequences [9]. Since the focus of this study was the PLN RΔ14 mutation that causes human cardiomyopathy, we carried out further staining for this construct in larger, more defined primary fibroblasts (**Figure 3B**). Here, we saw clear plasma membrane staining of the PLN RΔ14 which was comparable to the membrane staining of the two known plasma membrane proteins, namely the Na/K ATPase and the Na/Ca Exchanger.

**Figure 4:**
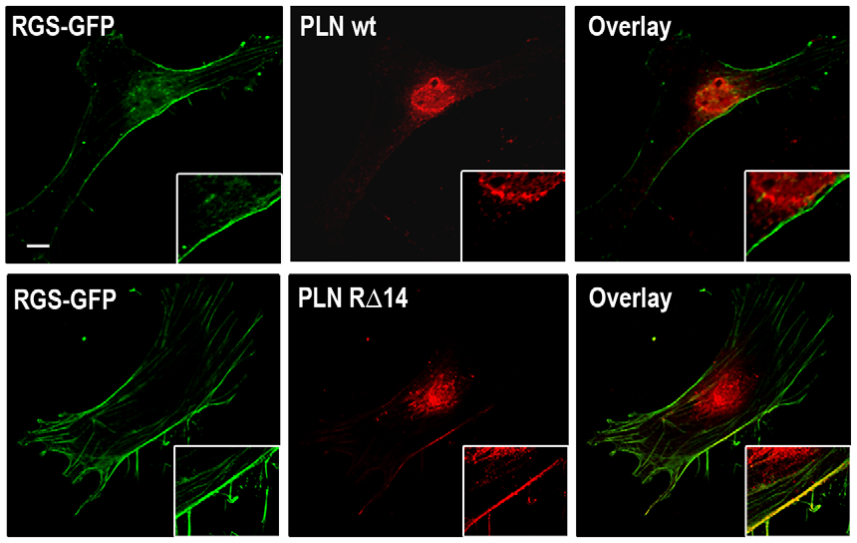
PLN arginine mutants co-localise with a known plasma membrane protein. Primary mouse fibroblasts were transiently co-transfected with a known plasma membrane localised RGS2-GFP construct (Δ1-71 amino acids) and either flag-tagged WT-PLN or the RΔ14 PLN construct, and visualized by confocal microscopy. WT-PLN did not show any plasma membrane staining and did not co-localise with the RGS2- GFP. However, RΔ14 PLN showed clear plasma membrane staining which was then confirmed with it co-localising with RGS2-GFP. (Scale bar, 10 µm)

In order to gain direct evidence that a mutated di-arginine motif causes mislocalization to the plasma membrane, we co-transfected primary fibroblasts with either wildtype NF-PLN and a RGS2-GFP (Δ1-71 amino acid) construct previously shown to be plasma membrane localised protein [28] (and a kind gift from Dr. Scott Heximer,U of Toronto) or PLN RΔ14 with RGS2-GFP. Co-staining of RGS2-GFP with wildtype PLN showed a distinct staining pattern for both proteins with RGS2-GFP localising to the plasma membrane and wildtype PLN to the ER (Figure 4) with no overlap between the two proteins. However, in contrast, PLN RΔ14 showed significant overlap with RGS2-GFP in the plasma membrane, providing further evidence of the mislocalization of PLN containing a mutated di-arginine motif (**Figure 4**).

### Detection of cellular proteins associating with PLN

**Figure 5:**
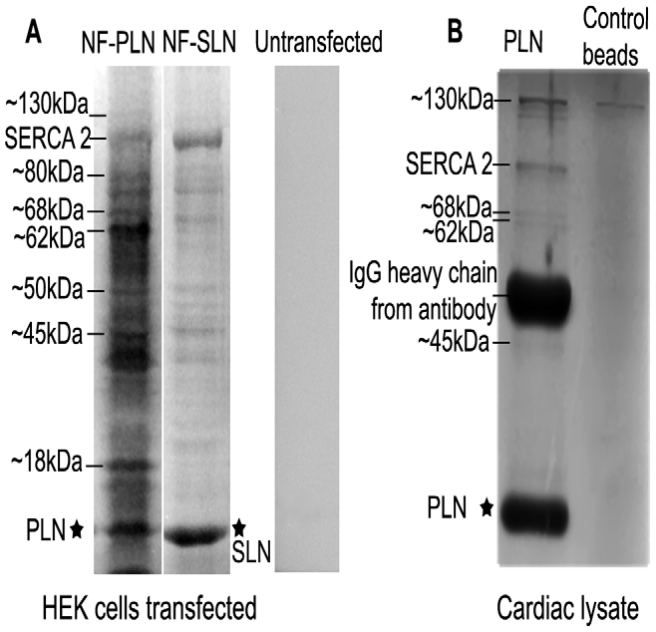
Identification of PLN interacting proteins. (**A**) HEK cells were transiently transfected with either tagged WT-PLN or tagged WT-SLN and then incubated with ^35^S-methionine for 6 hours. Untransfected cells were used as a control. IPs were performed with anti-flag antibody, prominent bands found in the PLN sample. (**B**) Coomassie stained SDS-PAGE gel of co-immunoprecipitated endogenous PLN from cardiac tissue. Lines indicate prominent proteins in sample, note molecular weights are similar to protein bands found in A.

In order to identify PLN interacting proteins, HEK cells were radiolabeled with ^35^S-methionine (Easytag express protein labelling mix, Perkin Elmer) for 2 hours, washed for 4 hours, and lysates collected 48 hours later. Radiolabeled lysates from NF-PLN transfected cells were subjected to immunoprecipitations using the anti-Flag antibody M2 and then subjected to SDS-PAGE and exposed onto autoradiographs (**Figure 5A**). Protein bands unique to NF-PLN and, therefore, representing potential binding partners of PLN, were seen at ∼130, 80, 68, 62, 50, 45 and 18 kDa. SERCA and PLN bands were evident at 110 kDa and ∼10 kDa, respectively. Control experiments were carried out with lysates from NF-SLN transfected cells and lysates from un-transfected cells that were processed in parallel. NF-PLN showed unique bands when compared to NF-SLN which can then be implicated as exclusive binding partners of PLN. Untransfected cells failed to show any specific enriched bands when subjected to SDS-PAGE.

Similarly, to identify proteins associating with endogenous PLN we carried out a second series of immunoprecipitations using the anti-PLN antibody 1D11 and fresh cardiac total muscle lysates. Protein bands were seen in a coomassie stained gel (**Figure 5B**) at molecular weights similar to those observed in the transfected HEK cells although not all the bands seen in the HEK cells were observed with the cardiac lysates, possibly due to differences in the immunoprecipitations carried out with HEK cells and cardiac lysates and the difference between the anti-Flag antibody M2 and the anti-PLN antibody 1D11.

### Identification of cellular proteins associating with PLN

**Figure 6:**
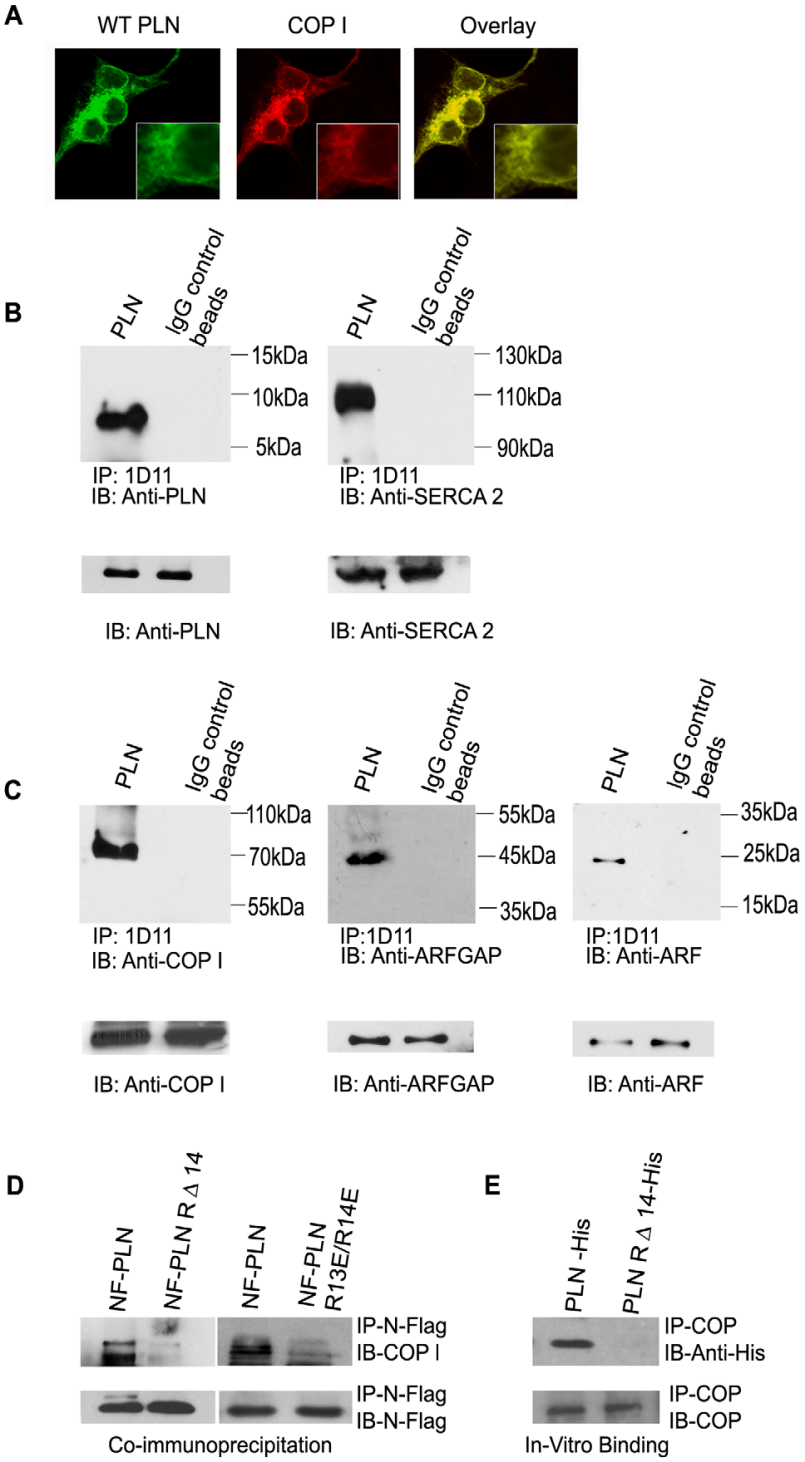
PLN binds to COP I coated transport vesicles in a di-arginine dependent manner. (**A**) Transfected HEK cells analyzed by confocal immunofluorescence experiments to detect PLN using anti-PLN antibody 1D11 and Fluor488, and the retrograde transport vesicle coat protein COP I detected using COPI antibody and Fluor633. (**B**) Co-immunoprecipitation of endogenous PLN from mouse heart tissue using anti-PLN antibody 1D11 followed by western blot anlaysis using 1D11 (left panel) or anti-SERCA2 antibody (right panel). IgG beads alone were used as negative controls. (**C**) Co-immunoprecipitation of PLN in wildtype mouse hearts was also analyzed for COP I (left panel), ARFGAP 1 (middle panel) and ARF (right panel); the essential core proteins in coatomer coated vesicles. IgG beads alone were used as negative controls. Lower panels are immunoblots showing equal loading of cardiac lysate to each reaction. (**D**) Co-immunoprecipitation performed using cell lysates from NF-PLN and NF-RΔ14 and NF-R13E/R14E transfected HEK cells. NF-PLN, NF-PLN RΔ14 and NF-R13E/R14E mutant were precipitated using anti-flag antibody and samples were then immunoblotted with COPI (upper panel) or anti-flag antibody (lower panel). The lower intensity signal of the RΔ14/COP I complex and R13E/R14E/COP I complex shows that binding of COP I is dependent on an intact di-arginine motif. (**E**) In-vitro binding assays were performed using purified proteins from cobalt beads (see methods). Purified His-COP I was combined with purified His-PLN or His-RΔ14. Immunoprecipitations were performed using COP I antibody. Immunoblots with anti-His antibody were used to identify His-PLN or His-RΔ14 at ∼5 kDa. Lower panel represent loading controls of COP which were identified using COP antibody at ∼130 kDa.

The recent findings that di-arginine motif binds to COP I [29] and that the position of the di-arginine motif is flexible throughout the protein [10], [30], led us to the hypothesis that PLN was involved in di-arginine mediated transport and prompted us to investigate the involvement of coatomer protein I (COP I) in PLN trafficking. PLN transfected HEK cells were analysed for co-localisation by immunofluorescence between PLN and COP I. When probed with PLN antibody 1D11 and COP I antibody (**Figure 6A**), the cells showed clear ER staining of both proteins and significant overlap of fluorescence, indicative of co-expression and general co-localization of these two proteins, as would be anticipated given the known localization of these proteins.

To verify binding of PLN and COP-I proteins, we performed co-immunoprecipitations by precipitating endogenous PLN from heart tissue with anti-PLN 1D11 and examining binding partners in the elution. First, we performed western blots to confirm PLN and known PLN binding partner SERCA2a and showed clear co-immunoprecipitation between these two interacting proteins (**Figure 6B**). Next, we determined that COP I binds with PLN, with a clear band observed in a COP-I immunoblot, at approximately 130 kDa (**Figure 6C**). Additional western blot analyses of the immunoprecipitated sample also identified two other proteins (ARFGAP1 and ARF1) that are known to be involved in COP I mediated transport [31]–[33]. Distinct bands were observed in these blots at the appropriate molecular weights: ARFGAP1 at ∼45 kDa; and ARF1 at ∼18 kDa (**Figure 6C**).

### Di-Arginine dependent COP I binding of PLN

To investigate the involvement of the di-arginine motif in COP I modulated transport cell lysates from cultures transfected with either NF-PLN, NF-PLN RΔ14 or NF-R13E/R14E were subjected to immunoprecipitations with anti-Flag antibody (**Figure 6D**). COP I co-immunoprecipitated with NF-PLN, which was detected by a western blot using COP I antibody. However, COP I showed a marked five-fold reduction in band intensity when co-immunoprecipitated with either NF-PLN RΔ14 or NF-R13E/R14E. This shows that PLN binding to COP I is dependent on the intact di-arginine motif.

### Direct Binding of PLN to COP I

To verify that PLN was binding directly to COP, we cloned COP, PLN and PLN RΔ14 into a His-tagged bacterial expression vector (pET28-MHL Vector; GenBank accession #EF456735), a kind gift from Dr Sirano Dhe-Paganon (U of Toronto). Proteins were purified individually using cobalt beads (see methods) and purified COP was incubated with either purified wild type PLN or purified PLN RΔ14 (**Figure 6E**). In vitro co-immunoprecipitation assays were performed with the COP antibody. Immunoblots using the anti-His antibody showed that wild type PLN was present in the COP-precipitated eluate however, PLN RΔ14 was not co-immunoprecipitated. These experiments provide evidence of direct binding between PLN and COP and provides further evidence that PLN binding to COP I is dependent on its intact di-arginine motif.

### Bioinformatic analysis of ER/SR proteins

**Figure 7:**
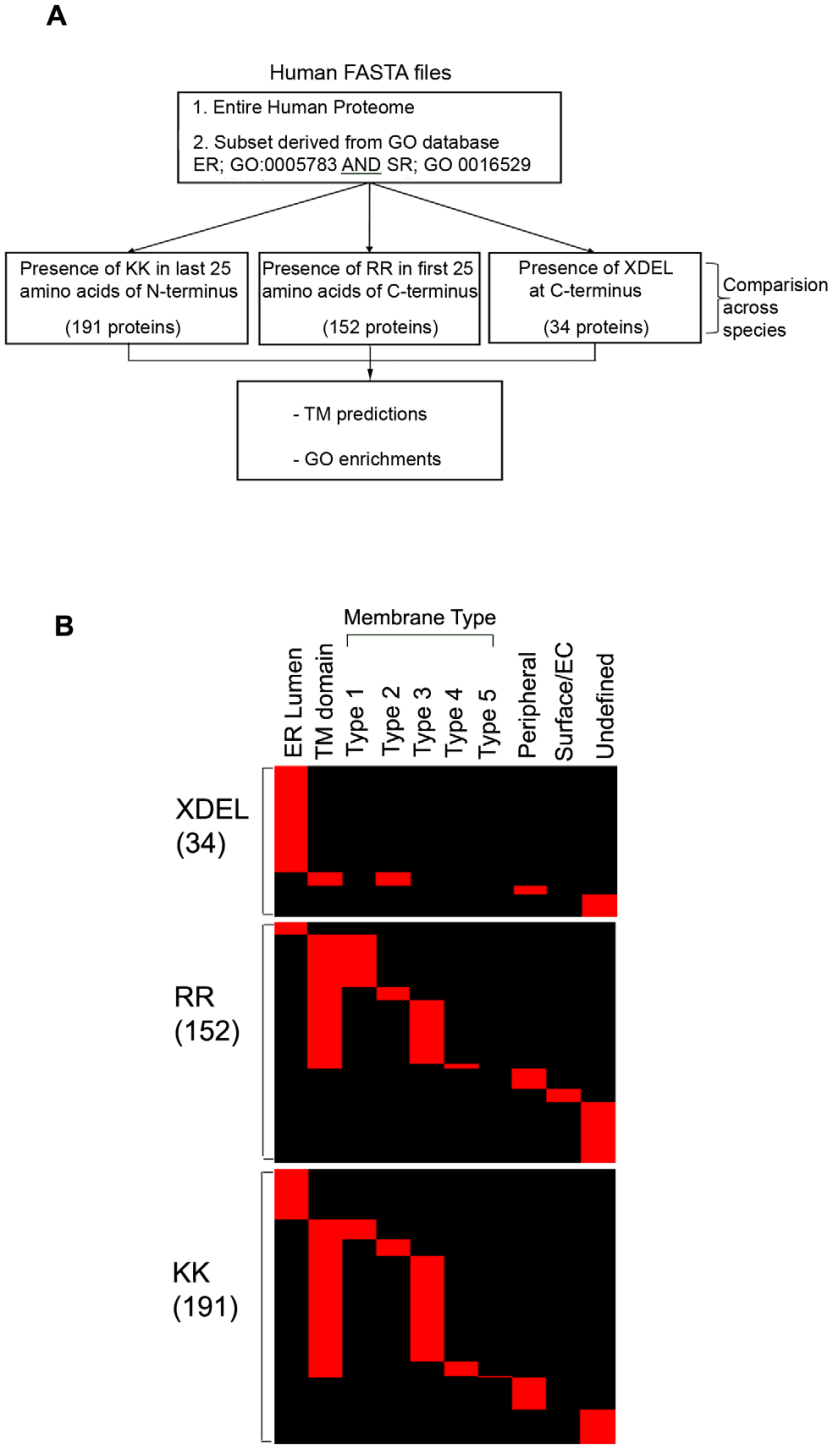
Heat map showing classification of membrane protein types. (**A**) Scheme of bioinformatic work flow (**B**) Proteins clusters obtained from human gene ontology sets ER (GO: 0005783) and SR (GO: 0016529) were characterized according to whether they were present in either the *ER lumen* or whether they contained a *transmembrane (TM) domain* based on annotations found in the ExPASy database (www.expasy.org). Proteins found to possess a TM domain were further classified according to the type of membrane protein; type 1 is a single pass protein with extracellular or luminal N-terminus; type 2 is a single pass protein with an extracellular or luminal C-terminus; type 3 is a multipass transmembrane protein; type 4 is a lipid chain-anchored membrane protein; and type 5 is a GPI-anchored membrane protein. Proteins were also defined as *peripheral proteins* and *cell surface or extracellular surface attached* proteins. *Undefined* proteins were those classified as having an ER/SR annotation, but no further detailed classification was available. Red indicates the presence of annotation and black indicates the lack of annotation.

Altogether, these findings then led us to investigate the potential that the di-arginine motif may be a common ER/SR retention motif that directs protein enrichment in the ER. From the human IPI protein sequence database we extracted all of the human ER (GO: 0005783) and human SR (GO: 0016529) annotated proteins, which represents all proteins that have a known and annotated ER and SR localization (**Figure 7A**). We then analyzed this list to identify proteins that contain: the motif RR within the proteins first 25 residues; the KK within the proteins last 25 residues; and proteins with the sequence XDEL at the C-terminus (**Table S1**). In total we found: 152 proteins containing the RR motif of which 84 had one or more transmembrane domains; 191 proteins containing the KK motif, of which 109 had one or more transmembrane domain and 34 proteins containing the XDEL motif, of which 24 were annotated to be found in the ER/SR lumen (**Figure 7B**). The enrichment in the RR and KK motifs in proteins with greater than one transmembrane domain when compared to the XDEL motif, which is enriched in proteins with no membrane domains, is as expected, since the XDEL motif mediates the retrieval of luminal ER proteins from the Golgi apparatus lumen proteins. Further annotation of these proteins into membrane protein types showed that the majority of proteins containing the KK and RR motifs were type 3 multi-membrane pass proteins (**Figure 7B**). Next, to characterize the *Cellular Component* ontology of the proteins identified in our bioinformatic screen containing the RR and XDEL motif we used the Gene Ontology schema and calculated significantly enriched GO-terms (**Table 1**). We found cellular component enrichments for the RR motif in ‘*cytoplasmic membrane bound vesicles’* and ‘*membrane bound vesicles*,’ both of which are components associated with vesicular protein trafficking. In contrast, the XDEL motif showed enrichments in ‘*endoplasmic reticulum lumen’*, ‘*membrane enclosed lumen*’ and ‘*organelle lumen*’, which are all components associated with soluble, lumenal proteins. The KK cluster did not contain any significant GO term enrichments.

**Table 1:** Gene Ontology enrichments (cellular components) of the RR and XDEL containing proteins.

Cellular Component for RR motif	Count	Ref. Count	Raw p-value	FDR p-value
nuclear membrane	18	57	1.66E-04	3.00E-02
cytoplasmic membrane-bounded vesicle	23	95	5.81E-04	3.93E-02
trans-Golgi network	9	18	6.29E-04	4.00E-02
membrane-bounded vesicle	23	98	8.46E-04	4.15E-02
cytoplasmic vesicle part	14	45	1.04E-03	4.15E-02
**Cellular Component for XDEL motif**				
endoplasmic reticulum lumen	31	99	1.557E-25	7.927E-23
membrane-enclosed lumen	31	151	1.106E-20	1.877E-18
organelle lumen	31	151	1.106E-20	1.877E-18
ER-Golgi intermediate compartment	8	42	0.00004703	0.001596
melanosome	6	24	0.0001293	0.003234

Cellular component enrichment of the RR and XDEL motif. GO-term cellular component analysis was carried out using ProteinCenter software suite (Proxeon, Denmark) on proteins identified in the bioinformatic screen as containing the di-arginine motif in the first 25 residues. The analysis shows significant enrichment of the di-arginine motif in cytoplasmic membrane and membrane bound vesicles in comparison to XDEL. This is enriched in ER and organelle lumens when compared to the reference count of total proteins found in the GO database with identifications of ER and SR. No GO-term enrichment was observed for the KK cluster.

**Figure 8:**
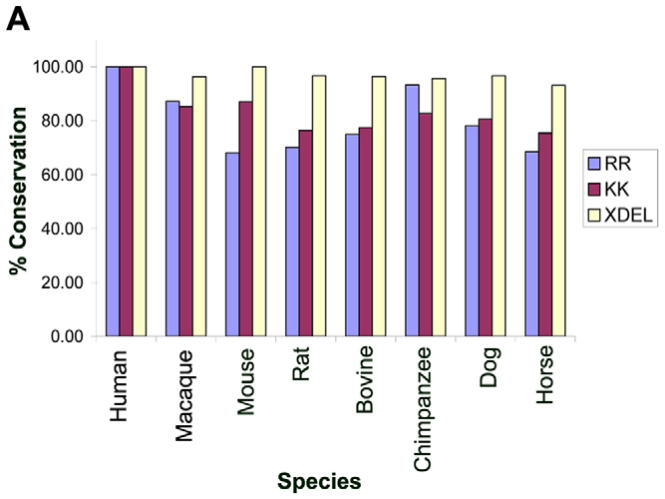
Percentage conservation of the di-arginine, di-lysine and XDEL motifs. (**A**) Graph representing the percentage of proteins identified in our bioinformatic screen that contain one or more transmembrane domains and retain the di-arginine or di-lysine motif or retain the XDEL motif and are lumenal proteins in a variety of species orthologues carried out using Basic Local Alignment Search Tool (BLAST). The proteins searched were those found in the human IPI protein sequence database that were annotated as ER (GO:0005783) and SR (GO:0016529) and containing the di-arginine motif in the N-terminal 25 residues or di-lysine motif in the C-terminal 25 residues and XDEL at the C-terminus.

Conservation of amino acid sequences across species infers that these sequences are either essential for viability and/or essential for interaction with other proteins [34]. We then used the Basic Local Alignment Search Tool (BLAST) to determine if the di-arginine motif identified in the bioinformatic screen in the first 25 residues were conserved throughout multiple species within the proteins containing one or more transmembrane domains. We found that the motif overall is preserved in 68% of mouse orthologues, 75% of bovine orthologues and 93% of chimpanzee orthologues (**Figure 8A** **and Table S2**). Comparable conservation was seen between protein orthologues containing one or more transmembrane domains with the known di-lysine motif in the last 25 residues and elevated conservation of the XDEL motif was seen in luminal proteins (**Figure 8A and 8B**). These findings further point to the importance of the di-arginine motif, given its conservation across multiple species.

### The di-arginine motif -a general ER retention motif?

In order to determine whether the di-arginine motif regulates a diverse set of proteins in the cell, we randomly selected two further membrane proteins identified in the bioinformatic screen as containing a di-arginine motif in the first 25 residues, and which had an available full length cDNA, and carried out site-directed mutagenesis of the di-arginine motif to glutamic acid.

**Figure 9:**
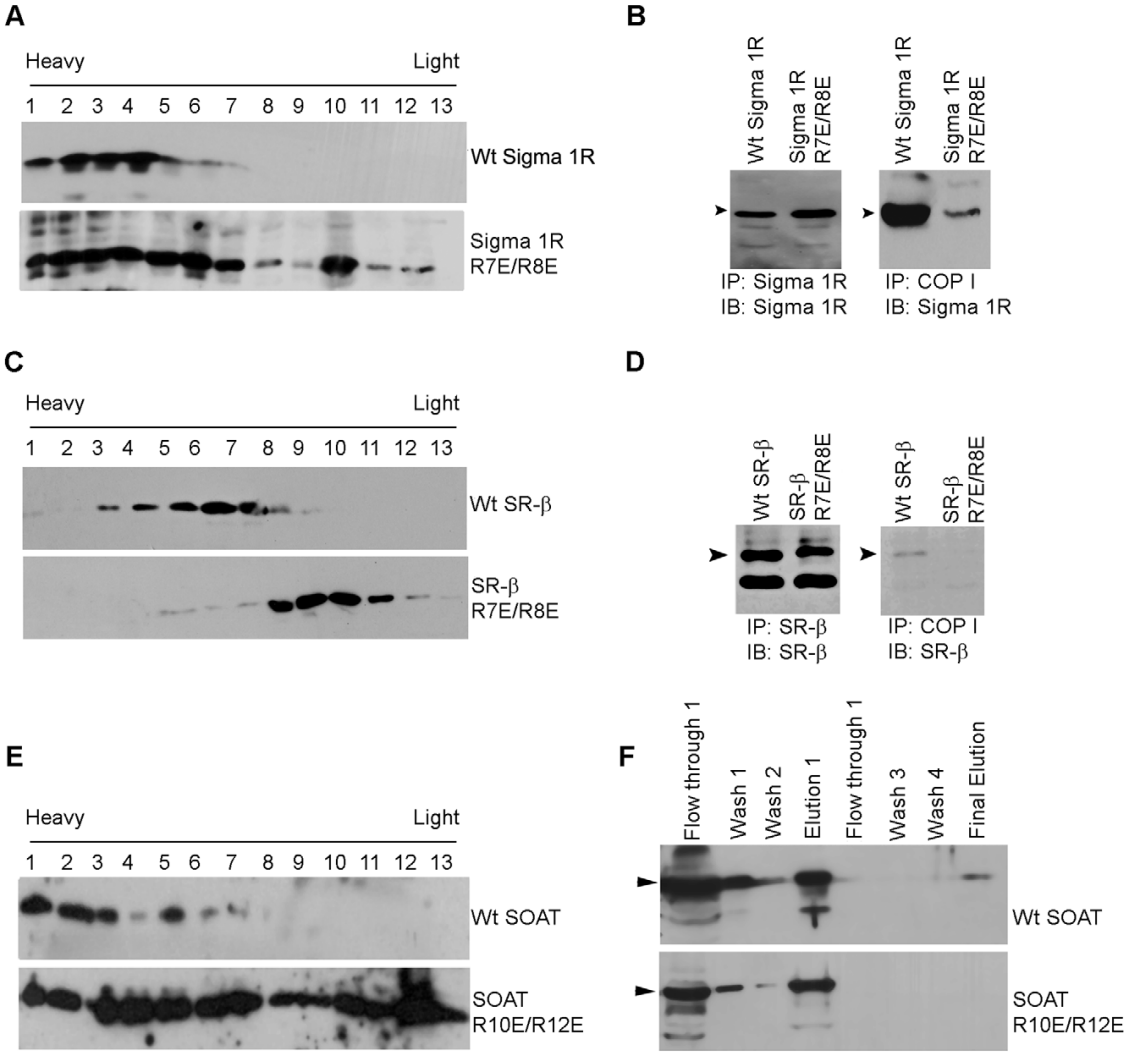
Mistargeting of other di-arginine mutants. (**A**) Subcellular fractionation of wild type Sigma 1R and a di-arg to di-glu mutant Sigma 1R construct. HEK cells were transiently transfected with tagged V5 Sigma 1R or tagged V5 di-arg to di-glu mutant Sigma 1R and cell lysates run on a continuous 20–60% sucrose gradient. Sigma1R was detected using the anti-V5 antibody. (**B**) IP performed using cell lysates from wild type V5 tagged Sigma 1R and V5 tagged di-arginine mutant Sigma 1R constructs transfected HEK cells. Proteins were immunoprecipitated using anti COP I antibody and then immunoblotted with anti V5 antibody (right panel); with equivalent loading of lysates (left panel). Arrows indicate Sigma 1R and mutant Sigma 1R. (**C**) Subcellular fractionation of wild type SR-β and di-arginine mutant SR-β construct. HEK cells were transiently transfected with V5 tagged SR-β or V5 tagged di-arginine mutant SR-β and cell lysates run on a continuous 20–60% sucrose gradient. SRB was detected using anti-V5 antibody. (**D**) IP performed using cell lysates from wild type V5 tagged SR-β and V5 tagged di-arginine mutant SR-β constructs transfected HEK cells and immunoprecipitated using anti COP I antibody and then immunoblotted with anti V5 antibody (right panel); with equivalent loading of lysates (left panel). Arrows indicate SR-β and mutant SR-β (**E**) Subcellular fractionation of wild type SOAT and di-arginine mutant SOAT construct. HEK cells were transiently transfected with tagged SOAT and di-arginine mutant SOAT and cell lysates run on a continuous 20–60% sucrose gradient and detected using anti-SOAT antibody (**F**) Tandem affinity purification of HEK cells transiently transfected with tagged SOAT and di-arginine mutant SOAT. Purification was carried out using streptavidin beads first and calmodulin beads second. Samples were then probed with antibodies to detect COP I, arrows indicate COP I (see methods for details).

The Sigma 1-type opioid receptor (Sigma 1R) is a broadly distributed integral membrane protein known to modulate various voltage-gated K^+^ and Ca^2+^ channels [35], [36]. Subcellular localization analysis by sucrose density fractionation of wild type sigma 1-type opioid receptor shows a tight localisation in heavy fractions 1–6, which is comparable to the ER markers seen in Figure 2. Mutation of both of the arginines in the di-arginine sequence (R7E/R8E) in Sigma 1R lead to a disrupted protein fractionation pattern with a significant amount of protein found in the lighter fractions, although protein was found across all fractions 1–12 (**Figure 9A**). Co-immunoprecipitation assays in HEK cells showed that COP I precipitated wild type Sigma 1R with a greater than two-fold band intensity than with the di-arginine mutated Sigma 1R (**Figure 9B**).

The β-subunit of the signal recognition particle receptor (SR β), is an ER membrane-embedded subunit of the signal recognition complex that is involved in the release of nascent polypeptide chains to the protein translocation machinery in the endoplasmic reticulum membrane [37], [38]. In Figure 9C, we show by sucrose density fractionation of WT SR β and an SR β di-arginine mutant (R7E/R8E). Wild type SR β localized in the heavy fractions 3–8, like the ER markers shown in Fig. 2, whereas the SR β di-arginine mutant localized predominantly in the light fractions 8–12 (**Figure 9C**) similar to the plasma membrane markers shown in Fig. 2. Co-immunoprecipitation assays also showed that COP I precipitated wild type SR β with a greater than two-fold intensity than the di-arginine mutated SR β (**Figure 9D**).

As a final candidate to test, we examined sterol-O-transferase (SOAT) which contains a xRxR motif within its first 25 amino acid residues. SOAT is an intracellular enzyme in the endoplasmic reticulum that covalently joins cholesterol and fatty acyl-CoA molecules to form cholesterol esters. It is a key enzyme in cellular cholesterol homeostasis [7]. Due to restricted cloning sites, SOAT cDNA (obtained from OriGene) was cloned into the Interplay Mammalian TAP vector, pCTAP (Stratagene) which contains a dual streptavidin binding peptide tag and a calmodulin binding peptide tag. Mutation of both arginine residues (R10E/R12E) in the motif resulted in the mislocalization of SOAT from the ER as monitored by sucrose density fractionation. Wild type SOAT localized in the heavy fractions 1–7, whereas the SOAT di-arginine mutant dispersed throughout the gradient (**Figure 9E**). Tandem affinity purification of wild type SOAT and di-arginine mutated SOAT was then carried out (see methods) and probed for COP I binding. Wild type SOAT purified with COP I but the di-arginine mutant purified SOAT did not copurify with COP I (**Figure 9F**).

## Discussion

### PLN is recycled via COP I mediated retrograde transport

Previously, we have shown that the RSYQY amino acid sequence at the C-terminus of SLN is vital in its ER/SR localisation [9]. The lack of this luminal sequence in PLN led us to pursue other mechanisms for PLN targeting to the ER/SR. In this study, we have shown that PLN retention in the ER is dependent on its R13/R14 di-arginine motif. The di-arginine motif had previously been implicated as an ER trafficking and retention signal. For instance, the correct assembly of multimeric plasma membrane proteins such as the K^+^(ATP)-sensitive channel and the GABA_B_ receptor is tightly coupled to the retention of their individual subunits within the ER [15], [39]. The K^+^(ATP) channel exists as an octameric complex composed of four pore-forming α subunits (Kir6.1/2), which have a RKR motif at the C-terminus and four sulphonylurea-binding β subunits (SUR1/2A/2B) with a RKR motif in the cytoplasmic loop. When correctly folded in its octameric form, the RKR motif in both subunits is masked and only then is the K^+^(ATP) channel exported to the cell surface. However, systematic removal of the RKR motif from the C-terminus of Kir6.2 resulted in significant expression at the cell surface [15], [39]. In the GABA_B_ receptor, the GB1 subunit contains a C-terminal RXR(R) motif and the surface expression of the functional receptor requires heterodimerization of GB1 and GB2 subunits [15], [39]. The retention signal is shielded by the interactions between GB1 and GB2 through the coiled-coil domains, thereby allowing the assembled GABA_B_ to traffic to the cell surface [15], [39]. These studies highlight the importance and versatility of the di-arginine motif in ER retention and provide a short amino acid sequence that is unrestricted by its positioning to the very C-terminal end of a protein, as is necessary with the di-lysine motif [10].

PLN expression has been observed outside of the ER, suggesting that it is not exclusively contained within the ER and indicating a requirement for further cellular trafficking mechanisms [40]. In this study, we have established a possible mechanism for the trafficking of PLN. Retrograde transport from the golgi to the endoplasmic reticulum has been well studied and is clearly mediated by COP I coated vesicles [10], [31], [41]. The minimal machinery necessary to form these retrograde transport vesicles has been shown to be only two proteins; coatomer protein and ARF1 with bound GTP [31]. Our co-immunoprecipitation of endogenous PLN from cardiac tissue showed that both of these proteins are bound to PLN. We also identified ARFGAP1, a GTPase-activating protein acting on ARF1 that has been shown to promote vesicle formation by functioning as a component of the COP I coat [32]. Our confocal microscopy showing complete overlap between COPI and PLN in the ER is entirely consistent with COPI mediated PLN trafficking. Together, our data provide the first evidence that PLN is recycled back to the ER via COP I mediated retrograde transport.

### Correct localization of PLN requires the intact di-arginine motif

We then investigated further the involvement of the di-arginine motif at the N-terminus of PLN. Our recent finding of a human deletion at Arg 14 (RΔ14) leading to hereditary cardiomyopathy gave us a relevant model to investigate the di-arginine motif. We found by confocal microscopy that mutation or deletion of either or both Arg 13 or Arg 14 caused mislocalization of PLN from the ER. Subcellular fractionation further enforced the finding that wildtype PLN fractionated with SERCA2a, a known ER marker, whereas PLN RΔ14, PLNR13E and the double mutant PLN R13E/14E showed some aspects of ER staining, much of these proteins fractioned with the Na/Ca exchanger, a known plasma membrane protein and with TGN46 a known trans golgi network protein. Co-immunoprecipitation further demonstrated that, in contrast to WT PLN, PLN RΔ14 did not co-purify with COP I. We confirmed direct binding between PLN and COP I by combining cobalt bead-purified proteins and showing direct interaction by co-immunoprecipitation of WT PLN and COP, but a lack of interaction between PLN RΔ14 and COP I. These data imply that COP I binds directly to the di-arginine motif in PLN, since mutation of this motif results in COP I not binding to PLN RΔ14 and its mislocalization to the plasma membrane.

It has been well established that PLN exists in an inhibitory monomeric form and as a less inhibitory or inactive pentameric form [42]–[45]. The normal pentamer to monomer ratio is 10∶1 and it has been determined that mutations that destabilise the pentamer increase the active monomer concentration inhibiting SERCA2a by altering the dissociation constant between the monomeric and pentameric forms of PLN causing inhibition by mass action [43], [46]. Previously, it was demonstrated that PLN RΔ14 mutation causes superinhibition of SERCA2a and that the mutation causes disruption in the stability of its pentameric form [4]. We also showed that coexpression of WT PLN together with PLN RΔ14 in HEK cells led to its immunoreactivity being localised to the ER [4]. This localization was very likely mediated due to mutant monomers being integrated into the WT PLN pentamer structure and maintained in the ER correctly. In this study however, we, have expressed PLN RΔ14 in the complete absence of any WT PLN, and the finding in this study that PLN RΔ14 is not recovered by COP I mediated retrograde transport and has becomes localized at the plasma membrane offers a mechanism by which the concentration of PLN is reduced in the ER and a possible explanation for the destabilisation of the pentameric form of PLN.

### The di-arginine motif can acts as a general ER/SR retention motif

The involvement of the di-arginine motif in PLN then led us to investigate the occurrence of the di-arginine motif in all ER and SR gene ontology (GO) annotated proteins. The finding that the frequency of the di-arginine motif is equivalent to the previously well established di-lysine motif and that the di-arginine motif proteins are localized in membrane-bounded vesicle and cytoplasmic membrane-bounded vesicle further supports the idea that the di-arginine motif is a major ER retrieval signal.

Protein sequences found in different species are orthologous if their amino acid sequences remain similar to each other (homologous) because they originated from a common ancestor. When orthology is unambiguous, proteins in the different species generally retain the same function, essentially when the function is critical to survival [34]. One reason for the maintenance of a motif through evolution is that it provides a necessary function, such as protein-protein interactions. The conservation of short peptide motifs is associated with transient transactions and conservation of larger domain motifs is associated with more stable protein-protein interactions [34], [47], [48]. We identified these ER and SR proteins in the human GO database which contain the di-arginine motif in a variety of species using Basic Local Alignment Search Tool (BLAST). The finding that the motif is conserved in 37 proteins in horse and up to 69 proteins in chimpanzee (**Figure 8**) further emphasizes the importance of the di-arginine motif.

It has been established that COP I binds directly to proteins bearing the di-lysine motif. Several proteins containing the di-lysine motif have been identified including ERGIC-53 and the KDEL receptor. Recycling of both proteins was efficiently inhibited when COP I function was blocked by microinjection of COP-I antibodies [49]. The di-arginine motif has also been shown to directly bind to the coatomer complex. The RSRR signal in the GB1 subunit of GABA_B_, for example was shown to specifically interact with COP I vesicles [39]. Further evidence of the di-arginine motif binding to coatomer protein was provided more recently by key mutations made to the α and β subunit of COP I, which although led to the formation of the coatomer complex had lost its ability to bind to the di-lysine motif [29].

In summary, we propose that PLN is retrieved from the golgi apparatus through the COP I retrograde pathway, and that COP I binds to the di-arginine motif in the cytoplasmic domain of PLN. A greater understanding of PLN protein synthesis and proper targeting in the cell may contribute novel approaches to deal with human PLN mutations that result in improper PLN protein synthesis, stability, or targeting. We also propose that the di-arginine motif may act as a general ER/SR retrieval motif and these finding warrants further investigation into the proper targeting of di-arginine motif containing proteins.

## Materials and Methods

### Cell Culture and Antibodies

HEK-293 cells (commercially bought from ATCC) were cultured in Dulbecco's modified Eagle's media H21 (Princess Margaret Hospital, Toronto) supplemented with 10% fetal calf serum and 1% non-essential amino acids. The culture and transfection of HEK-293 cells has been described in pervious publications [7], [45]. Primary mouse fibroblasts were isolated from differential platting of day 1 mouse hearts and cultured as previously described [50]. Briefly, mouse hearts were digested with 50 U/ml collagenase typeII (Worthington Biochemicals, Lakewood, NJ) and 0.5 mg/ml trypsin (Invitrogen Canada Inc., Burlington, ON, Canada) in calcium- and bicarbonate-free Hanks' buffer with HEPES. Dissociated cells were collected every 3 to 5 min. Fibroblast were collected by differential plating by utilising the ability of the fibroblasts to attach to the tissue culture plates faster than other cell types. Primary fibroblasts were then cultured in Dulbecco's modified Eagle's media H21 (Princess Margaret Hospital, Toronto) supplemented with 10% fetal calf serum and 1% non-essential amino acids. PLN antibody 1D11 was a kind gift from Dr Robert G. Johnson. Other antibodies were obtained commercially: FLAG antibody (M2)(Sigma), His antibody (Qiagen), COP I β subunit (Abcam), NCX (Santa Cruz), Na/K ATPase (Hybridoma Bank), SERCA2a (Affinity Bioreagents), ERGIC 53 (Sigma), Calnexin (Abcam), Calreticulin (Affinity Bioreagents), PDI (Calbiochem), Estrogen Receptor beta (Abcam), ARFGAP (Santa Cruz), ARF1 (Abcam), SOAT antibody (Abcam), TGN46 (AbD Serotec) a kind gift from Dr Walter Kahr (U of Toronto), Alexa mouse Fluor488 and rabbit Fluor633 (Invitrogen).

### Expression plasmids and mutagenesis

Mutagenesis and deletion of cDNA encoding rabbit phospholamban (PLN) containing a deletion of the arginine residue at position 14 has been described in previous publications [4]. Additional mutants generated in this study were the N-flagged PLN with R13E mutation, R13A, R13E/R14E double mutant. The primers were as follows: R13E mutation sense

5′ACTCGCTCTGCTATAGAAAGGGCCTCAACCATTGAA-3′ and antisense

5′TTCAATGGTTGAGGCCCTTTCTATAGCAGAGCGAGT-3′: R13A mutation sense

5′ACTCGCTCTGCTATAGCAAGGGCCTCAACCATTGAA-3′ and antisense

5′-TTCAATGGTTGAGGCCCTTGCTATAGCAGAGCGAGT-3′:

R13E/R14E mutation sense

5′ACTCGCTCTGCTATAGAAGAGGCCTCAACCATTGAA-3′, and antisense

5′TTCAATGGTTGAGGCCTCTTCTATAGCAGAGCGAGT-3′: RΔ14 mutation sense

5′ACTCGCTCTGCTATAAGAGCCTCAACCATTGAA-3′ and antisense

5′TTCAATGGTTGAGGCTCTTATAGCAGAGCGAGT-3′.

Underlined nucleotide indicates residues changed from wild type PLN in generating mutation (See Figure 1C)

Sigma 1R and SR β cDNA was purchased from Open Biosystems in the pDONR223 Gateway Entry Vector and then cloned into the Gateway pEF-Dest51 Vector (Invitrogen) which contains a V5 and 6His tags. The di-arginine mutations in Sigma 1R were generated using the following primers for the double mutant R7E/R8E: 5′-TGG GCC GTG GGC GAG GAG TGG GCG TGG GCC-3′ sense and 5′-GGC CCA CGC CCA CTC CTC GCC CAC GGC CCA-3′ antisense primers. The di-arginine mutations were generated in SR β using the following primers for the double mutant R7E/R8E: 5′-TCC GCG GAC TCG GAA GAG GTG GCA GAT GGC-3′ sense and 5′-GCC ATC TGC CAC CTC TTC CGA GTC CGC GGA-3′ antisense primers. SOAT-1 cDNA was purchased from Origene Technologies (Rockville, MD) in a pCMV6 plasmid vector and then inserted into the Interplay Mammalian TAP vector pCTAP from Stratagene. Site directed mutagenesis of the SOAT-1 was preformed using the following primers for the double mutant R10E/R12E: 5′-AAG ATG TCT CTA GAA AAC GAG CTG TCA AAG TCC-3′ sense 3′GGA CTT TGA CAG CTC GTT TTC TAG AGA CAT CTT 5′ anti-sense primers. Underlined bases indicate bases that were changed from wild type.

### Sucrose Gradient Fractionation

HEK-293 cells transfected with PLN or the PLN di-arginine mutants (PLN RΔ14, PLN R13E, PLN R13E/R14E) were harvested 48 hours after transfection. Cells were resuspended in a low ionic strength lysis buffer (10 mM Tris-HCl pH 7.5 and 0.5 mM MgCl_2_) and homogenised with 40 strokes in a dounce homogeniser at 4°C. Buffer A (250 mM sucrose, 50 mM Tris-HCl (pH 7.4), 1 mM PMSF, 20 µg/ml aprotinin) was added to further help solubilisation and a further 20 strokes applied in the dounce homogeniser. The sample was then centrifuged for 20 min at 14,000 *g*, and the supernatant was collected and layered on top of a 20–60% linear sucrose gradient made up in 10 mM Tris-HCl (pH 7.6), 10 mM EDTA and protease inhibitors (Roche). Samples were centrifuged at 100,000 g for 20 hrs in a SW40Ti swinging bucket rotor. Fractions (750 µl) were collected from the bottom of each gradient and total protein concentration calculated by using Bradford Reagent (Sigma).

### Immunofluorescence

HEK-293 and fibroblast cells were grown on glass chamber culture slides (BD-Falcon) and transfected using lipofectamine (Invitrogen) following the manufacturers instructions. After 48 hours the cells were washed in phosphate buffered saline (PBS) and fixed in 2% paraformaldehyde in PBS (pH 7.0). Non-specific interactions were suppressed with 5% fetal bovine serum (FBS) in permeabilization buffer (0.2% tween-20, 0.5% Triton X-100 in PBS pH 7.0) for 30 minutes and then samples were incubated with primary antibodies anti-PLN 1D11 and anti-COP I in permeabilization buffer. Cells were then washed 3 times with PBS and incubated with either Alexa Fluor 488 for PLN or Alexa Fluor 633 for COP I. Images were collected by using a Leica DM IRBE inverted microscope equipped with a Leica TCS SP laser scanning confocal system. Spectra for Alexa Fluor 488 were collected by excitation at λ488 and emission collected between λ490–λ510 and spectra for Alexa Fluor 633 were collected by excitation at λ633 and emission between λ640–λ670.

### Immunoblot and immunoprecipitation

Immunoblot analyses were performed using standard SDS-PAGE chemiluminescent procedures [9]. Immunoprecipitations were carried out using Protein G-Sepharose beads (Pierce) [9]. Briefly, a post-nuclear fraction was obtained from cells transfected with either PLN-WT, PLN RΔ14, PLN R13E/R14E, Sigma 1R-WT, SRβ-WT, SOAT-WT, Sigma 1R R7E/R8E, SRβ R7E/R8E, and SOAT R10E/R12E. Cells were collected in PBS containing 5 mM EDTA and the pellet was washed once in PBS. The cells were then lysed with 30 strokes in a tight- fitting glass dounce homogeniser in lysis buffer (250 mM sucrose, 50 mM Tris-HCl pH 7.6, 1 mM MgCl, 1 mM DTT, 1 mM PMSF). The lysate was then cleared by centrifugation for 15 min at 2600 rpm at 4°C. To allow antibody-protein complex formation, the cleared lysate was incubated at 4°C under continuous rotation with antibody in binding buffer (140 mM NaCl, 8 mM NaPO4, 2 mM KPO4, 14 mM KCl pH 7.4) and 0.1% Triton-X100, 0.01% BSA for 2 hours. Protein G-Sepharose beads were blocked in 0.1% BSA in binding buffer for 2 hours. The beads were then pelleted and added to protein sample and allowed to rotate overnight at 4°C. Samples were washed 5 times and then eluted in 0.1 M glycine pH 2.3. Immunoprecipitation of endogenous PLN from mouse cardiac muscle was carried out using the Seize-X Protein A Immunoprecipitation Kit (Pierce) following the manufacturers conditions. Antibody-protein G complexes were formed at 4°C for 4 hours, washed extensively, then incubated with post nuclear extract overnight.

### *In-vitro* binding assay

The cDNAs of COP (Open Biosystems), PLN and PLN RΔ14 were cloned into the His-tagged bacterial vector pET28-MHL Vector (GenBank accession EF456735). The His-tagged proteins were expressed in 1 liter of 2YT overnight at 37°C under continuous shaking and protein production was induced by the addition of IPTG. Bacterial pellets were centrifuged at 6000 g and resuspended in binding buffer (50 mM Tris-HCl, pH 8.0, 200 mM NaCl, 1 mM β-mercaptoethanol and 2 mM imidazole, pH 8.0) and incubated with cobalt beads overnight at 4°C under continuous shaking. The beads were then washed 3 times for 20 minutes each with wash buffer (50 mM Tris-HCl, pH 8.0, 200 mM NaCl, 1 mM β-mercaptoethanol and 10 mM imidazole, pH 8.0). Proteins were then eluted in elution buffer (50 mM Tris-HCl, pH 8.0, 200 mM NaCl, 1 mM β-mercaptoethanol and 500 mM Imidazole, pH 8.0). Purified COP I and PLN or PLN RΔ14 were incubated together and immunoprecipitation was carried out as described above.

### Tandem Affinity Purification

Due to cloning difficulties, SOAT cDNA (OriGene) was cloned into the Interplay Mammalian TAP vector, pCTAP (Stratagene) which contains a dual streptavidin binding peptide tag and a calmodulin binding peptide tag. Purification was carried out as recommended by the manufacturer in the Interplay TAP purification kit (Stratagene). In short, the protein extract was added to the washed steptavidin resin and rotated for 2 hours at 4°C to allow the tagged proteins to bind streptavidin via the streptavidin binding protein (SBP) tag and washed three times in wash buffer (Stratagene). For further purification, bound proteins were bound to calmodulin beads for 2 hours at 4°C via their calmodulin binding protein (CBP) tag. Beads were then washed and eluted in elution buffer (Stratagene) and probed for SOAT and COP, using the antibodies defined above.

### Bioinformatics

ER retention motifs KK and RR within the first 25 residues and ER retrieval motif XDEL in the C-termini of proteins were retrieved from the human IPI protein sequence database from proteins with GO identifications as ER (GO:0005783) and SR (GO:0016529). Protein orthologues were then compared over multiple species using BLAST (http://blast.ncbi.nlm.nih.gov/Blast.cgi). Data analysis and annotation of cellular components were carried out using ProteinCenter software suite (Proxeon, Denmark) as described previously [51].

## Supporting Information

Table S1 The list of proteins that contain the motif RR within the proteins first 25 residues, KK within the proteins last 25 residues and proteins with the sequence XDEL at the C-terminus, extracted from the human IPI protein sequence database from all the human ER (GO: 0005783) and human SR (GO: 0016529) proteins, which represents all proteins that have a known and annotated ER and SR localization.
(0.13 MB XLS)

Table S2 A table of percentage conservation of the di-arginine, di-lysine and XDEL motifs. Table showing the percentage of proteins identified in our bioinformatic screen that contain one or more transmembrane domains and retain the di-arginine or di-lysine motif or retain the XDEL motif and are lumenal proteins in a variety of species orthologues carried out using Basic Local Alignment Search Tool (BLAST). Numbers in brackets represent actual numbers.
(0.02 MB XLS)
